# Molecular Typing of *Acanthamoeba* Using Mitochondrial rDNA Spacers

**DOI:** 10.3390/microorganisms13102285

**Published:** 2025-09-30

**Authors:** Daniele Corsaro

**Affiliations:** CHLAREAS, 12, rue du Maconnais, 54500 Vandoeuvre-lès-Nancy, France; corsaro@gmx.fr

**Keywords:** *Acanthamoeba*, genotype, molecular typing, mitochondrial DNA, mitochondrial spacer, tRNA

## Abstract

*Acanthamoeba* is a widespread free-living amoeba known as an opportunistic parasite of humans and other animals. It comprises several species, whose characterisation relies currently on the analysis of 18S rDNA sequences, recognising more than twenty genotypes; however, the distinction between closely related lineages remains unclear. In this study, the spacer region between the mitochondrial large and small subunits of rRNA genes was analysed for its usefulness as a marker for molecular typing. Previous studies have shown that the mitochondrial spacer contains a group of five transfer RNA (tRNA) genes, and that its length and sequence vary considerably between strains. A total of forty-two mitochondrial spacers were examined here, including twenty-five newly recovered sequences, from ten genotypes covering the three morphological groups of *Acanthamoeba*. The results showed that lineage-specific profiles can be defined for morphological groups 2 and 3 species (MG2 and MG3), with phylogenetic analysis consistent with that of rDNA, allowing for strain identification at the subtype level. In addition, morphological group 1 (MG1) species have a different tRNA gene arrangement distinguishing them from the others. Mitochondrial spacers therefore appear to be promising phylogenetic markers for the molecular typing of *Acanthamoeba*.

## 1. Introduction

*Acanthamoeba* (Amoebozoa, Discosea, Centramoebida, Acanthamoebidae) is a genus of free-living amoebae, distributed worldwide in both aquatic and terrestrial environments. Its life cycle includes an active trophozoite feeding on bacteria and other microbes, and a highly resistant, double-walled dormant cyst. It deserves special attention because it can act as an opportunistic parasite in humans and other vertebrates, primarily causing amoebic keratitis (AK), leading to vision loss, and fatal granulomatous amoebic encephalitis (GAE), often due to the hematogenous spread of a pulmonary or skin infection [1,2]. Disease severity can vary considerably depending on the patient’s health status and the pathogenicity of the amoeba strain. Indeed, *Acanthamoeba* exhibits high genetic and pathogenic variability, with some species appearing more virulent than others, although a clear relationship has not been established. Furthermore, *Acanthamoeba* frequently harbours endosymbionts, including pathogens such as *Legionella*, thereby contributing to their dissemination [3].

*Acanthamoeba* comprises several species, divided into three morphological groups (MG1 to MG3) based on cyst characteristics. MG1 species have a stellate endocyst with a well-separated ectocyst, while MG2 species may have a stellate, polygonal, or nearly spherical endocyst with a usually wrinkled ectocyst, and MG3 species have a round endocyst with a usually smooth ectocyst [4]. *Acanthamoeba* lineages are currently reorganised into more than twenty genotypes (T1 to T23) by molecular phylogenetic analysis based on the nuclear small subunit (SSU) ribosomal RNA gene (18S rDNA). The molecular approach allows the recovery of both classical and novel species, also revealing some inconsistencies based on previous morphological identifications, which have led to numerous strain misassignments [5,6]. Analysis of 18S and large subunit (LSU) nuclear rDNA sequences indicates greater diversification of *Acanthamoeba*, with more undescribed lineages that could represent new genotypes or even new genus-level taxa [7]. However, the vast majority of strains isolated from clinical and environmental samples belong to the T4 genotype, a large group comprising closely related strains and including the type strains of several MG2 species. Improving the resolution of phylogenetic relationships within closely related lineages is clearly useful for taxonomic purposes, but it is also useful for more reliable diagnosis and epidemiological investigation. In the case of T4, various nuclear and mitochondrial SSU rDNA subtypes have been defined, with good consistency between them [6,8]. They are based on complete sequences, approximately 2300–2600 or 1500 bp for the nuclear or mitochondrial SSU, respectively, and currently comprise eight nuclear subtypes (T4A to T4H) and ten mitochondrial subtypes (T4a to T4j) [6,8,9,10]. Promising results have also been obtained using the rapidly evolving internal transcribed spacer (ITS) region of the nuclear rDNA operon [11,12,13]. In almost all eukaryotes, this region contains the 5.8S rRNA gene, which is separated from the SSU and LSU rRNA genes by ITS1 and ITS2, respectively. It is used as a whole to distinguish, for example, species and genotypes of *Naegleria* (Discoba, Heterolobosea), a potentially pathogenic amoeboflagellate [14], whereas variations in the eukaryotic core secondary structure of ITS2 correlate with lineage separation [15].

Another potentially useful marker for differentiating *Acanthamoeba* strains could be the spacer between the mitochondrial LSU and SSU rRNA genes, which closely resembles the bacterial spacer except that the rRNA genes are inverted. In prokaryotes, the ITS often includes transfer RNA (tRNA) genes, while the homologous part of 5.8S is included in the 5’ end of the LSU [16,17], and the ITS region is increasingly used for the identification of bacteria [18,19]. In the Neff strain, now *Acanthamoeba terricola* [20], this region contains five tRNA genes [21]. By analysing the mitochondrial ITS of sixteen other strains belonging to four genotypes (T1, T2, T3, T4), Ledee and Byers [22] found the same tRNA genes, in the same order, but also considerable heterogeneity in the sequence and length of the intergenic parts. The authors concluded that ITS sequences are often difficult to align and therefore have little phylogenetic utility. In this study, the mitochondrial ITS sequences of twenty-five additional strains belonging to eight genotypes were retrieved from the GenBank database and analysed, allowing the identification of lineage-specific profiles.

## 2. Materials and Methods

Mitochondrial spacers flanked by ~120 nucleotides of the 3’ and 5’ ends of the LSU and SSU rRNA genes, respectively, were extracted from *Acanthamoeba* genomes available in GenBank, using the mitochondrial DNA (mtDNA) sequence of *A. terricola* strain Neff (U12386.1:4994–5883) [21] as a query in BLAST searches available at: [https://blast.ncbi.nlm.nih.gov] (accessed on 17 August 2025). The same search was performed on a selected set of Sequence Read Archive (SRA) data. For three strains (BCP, Linc-AP1, TN), the sequences were extracted from the available mitochondrial genomes. The remaining mitochondrial spacers analysed were those obtained by Ledee and Byers [22] (Table S1).

For the new spacers obtained, the various tRNA genes were identified by comparing with the already available sequences or by searching for the specific anticodons and modelling the secondary structures. For spacers from MG2 and MG3 species, a multiple alignment was prepared using MAFFT (L-INS-I option) and visually verified with BioEdit to ensure the correct positioning of the tRNA genes. To strengthen the analysis, 20 nt from the ends of both rRNA genes were included. In two cases, the tRNA genes for alanine (*trnA*) and for proline (*trnP*) overlapped by one or three nucleotides, and they were rearranged in the alignment as single elements. Molecular phylogenetic analyses were performed as described previously [23,24] using maximum likelihood (ML; GTR, *Γ* + I:4 model), distance (Neighbour-Joining, NJ, Kimura 2-P), and maximum parsimony (MP), with 1000 bootstraps.

## 3. Results

### 3.1. Acanthamoeba Species MG2/MG3 and MG1 Differ in the Arrangement of tRNA Gene Clusters

Sequences of the mitochondrial spacer flanked by the LSU and SSU ends were recovered from the genomes of twenty-three MG2 and MG3 strains belonging to six genotypes (T3, T4, T5, T11, T13, T22), as well as two MG1 strains, *Acanthamoeba astronyxis* (T7) and *Acanthamoeba byersi* (T18). For MG2 and MG3 strains, the spacer lengths range from approximately 480 nt for *Acanthamoeba lenticulata* (T5) to 1360 nt for *Acanthamoeba palestinensis* (T2), which has the particularity of having a duplicated tRNA gene for methionine (*trnM*) [22]. For both MG1 species, *A. astronyxis* and *A. byersi*, shorter spacers of similar length, 438 and 519 nt, respectively, were recovered.

**Figure 1:**
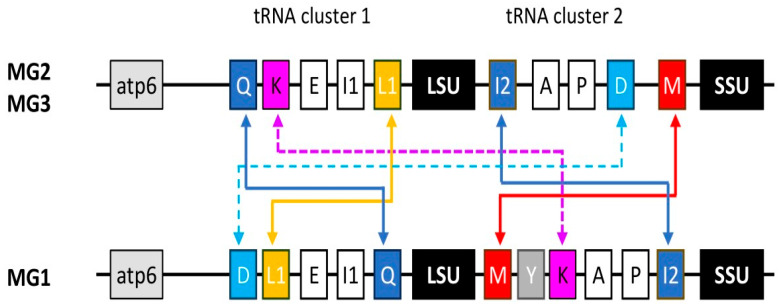
Mitochondrial tRNA gene clusters. Schematic drawing (not to scale) of the *Acanthamoeba* mtDNA region spanning the LSU and SSU rRNA genes, as well as the two tRNA gene clusters located upstream of the LSU rDNA (cluster 1) and in the spacer (cluster 2). The tRNA genes are indicated by the conventional one-letter code, with those showing different arrangements between the MG2/MG3 and MG1 species colour-coded. The solid and dotted arrows indicate rearrangements within or between the two gene clusters, respectively.

All spacers in MG2 and MG3 strains contain the same tRNA genes (tRNA cluster 2), in the same order as previously reported [21,22]: *trnI2* (isoleucine), *trnA* (alanine), *trnP* (proline), *trnD* (aspartic acid), and *trnM* (methionine). This arrangement was also observed in *Acanthamoeba culbertsoni* (T10), whose sequence was however excluded from the analysis due to not having sequenced gaps in the contig. In contrast, a different situation was observed for MG1 strains. *A. byersi* has six tRNA genes, consisting of *trnM*, *trnY* (tyrosine), *trnK* (lysine), *trnA, trnP*, and *trnI2*, and *A. astronyxis* has the same arrangement, except that it lacks *trnY*. Compared to MG2 and MG3, two genes (*trnM* and *trnI2*) have reversed their position, and another (*trnK*) appears to have replaced *trnD*, but at a different position. In the Neff strain, *trnK* is part of a group of five other tRNA genes located just upstream of the LSU rRNA gene (tRNA cluster 1) [25]. The same arrangement is found in the available mitogenomes of other T4 strains, as well as by analysing genomic data from the other genotypes studied. However, for this region as well, *A. byersi* and *A. astronyxis* exhibit a different arrangement, with the reversed position of two genes, *trnQ* (glutamine) and *trnL1* (leucine), and the presence of *trnD*, which, in MG2/MG3 species, is located in the spacer (Figure 1).

Although the analysis is not exhaustive due to the limited number of strains and genotypes, it can be assumed that the arrangement of tRNA clusters 1 and 2, as observed in the Neff strain, is highly conserved in the MG2 and MG3 species. On the other hand, their rearrangement in *A. byersi* and *A. astronyxis* would allow a clear distinction between MG1 species and other *Acanthamoeba*, as already observed with other genetic markers. No data are available for other MG1 species. However, the order inversion between *trnA* and *trnI2* has already been observed by Ledee and Byers [22] through preliminary results on *Acanthamoeba comandoni* (T9). The different arrangement of the tRNA genes could explain the difficulties encountered in completing the sequence.

### 3.2. Mitochondrial Spacers of Acanthamoeba Species MG2/MG3 Correlate with Lineage-Specific Profiles

In their previous study [22], Ledee and Byers compared spacers from *A. terricola* (Neff strain) and sixteen other strains, mainly belonging to the T4 genotype, noting high variability in length and sequence for the regions between the tRNA genes. In particular, they found that only sequences from strains known to be strictly related based on mitochondrial SSU (mtSSU) rDNA data could be reliably aligned, but only partially. The longer intergenic region, separating *trnD* and *trnM*, was often too divergent, and the spacer appeared of little phylogenetic interest. Similar high variability was also found for the additional twenty-three spacers reported here; however, differences in spacer size and region length between tRNA genes make sense when strains are ordered according to mtSSU type/subtype, particularly for T4 strains. In fact, strains of the same type/subtype have spacers and intergenic parts of similar length (Table 1).

Also, sequences of a same subtype differ from each other by a few nucleotides and/or indels, while those of distinct subtypes show greater variations but are still alignable. Furthermore, alignment is also possible for large portions of the *trnD*–*trnM* region. It is likely that the discrepancies with Ledee and Byers’s study can be explained by the larger number of sequences analysed here and the use of a different, and probably more efficient, alignment program.

**Figure 2:**
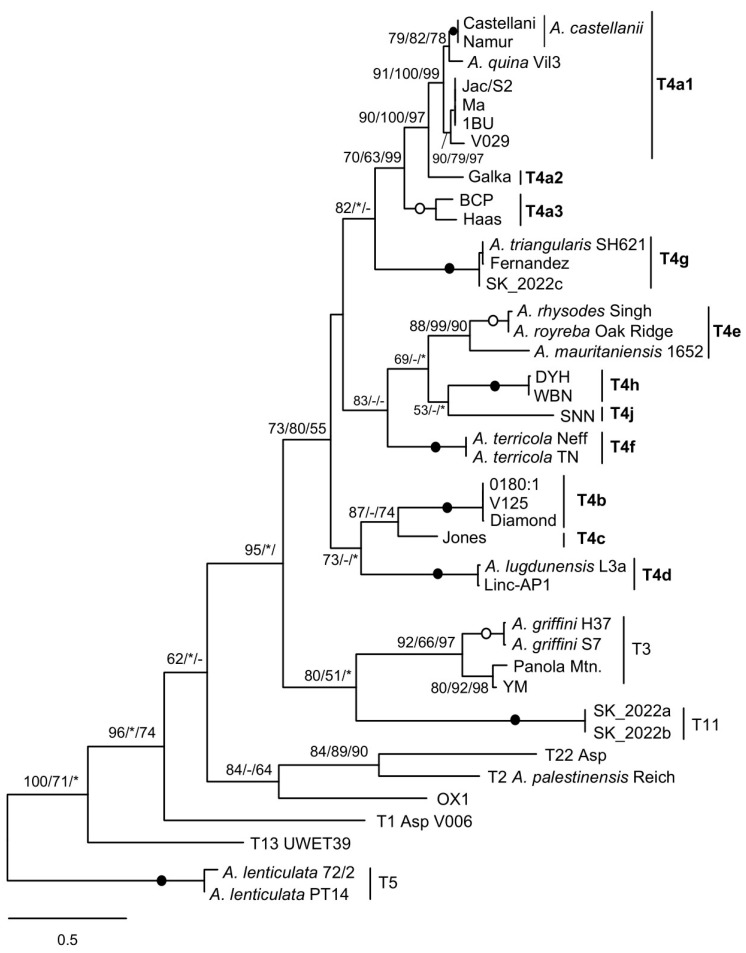
Molecular phylogeny based on mitochondrial rDNA spacer sequences. For T4A strains, mtSSU subtypes are indicated in bold. Spacers from T5 strains were used to root the tree. At the nodes, bootstrap values (BV) are shown for ML/NJ/MP, with filled and open circles for 100 or >95% BV support with all methods. Asterisk, BV < 50%; hyphen, node not recovered.

Phylogenetic analysis of the spacer sequences resulted in a tree in which the T4 strains are grouped together and have the T3 and T11 strains as their sister group, consistent with results obtained with other genetic markers. It is noteworthy that the branching pattern of T4 sequences fully recognises the mtSSU subtypes as clearly distinct and strongly supported lineages, although the relationships between them are less well established (Figure 2).

In particular, several previously proposed groups [6], such as the Diamond group (T4b), Haas/BCP (Haas group) (T4a3), or *Acanthamoeba lugdunensis*/Linc-AP1 (T4d), are well recovered, as is the sister relationship between *A. castellanii* and *Acanthamoeba quina*.

An unexpected result is the clustering of the *A. palestinensis* (T2) sequence with that of genotype T22 instead of that of strain OX1 CCAP 1501/3C, because both belong to the *A. palestinensis* group (T2/T6 clade). The *A. palestinensis* spacer has a *trnM* duplication [22], but this is unlikely to be the cause, since the same branching was found by removing the extra *trnM* (not shown). *A. palestinensis* and OX1 differ at two or three sites in the five tRNA genes. In pairwise comparisons, the *A. palestinensis* spacer without the additional *trnM* is 68.8% similar to that of OX1, and both spacers are weakly related (~46%) to that of *Acanthamoeba* T22. The value between *A. palestinensis* and OX1 is similar to that observed between spacers of distantly related T4 subtypes (~65%), whereas between more closely related subtypes, the values are > 80%. This seems consistent with the assignment of OX1 to its own type within the *A. palestinensis* group, as previously suggested [13,23], and analysing more strains should resolve the relationship found here, which is likely an artifact.

### 3.3. RNA Editing

Most *Acanthamoeba* mitochondrial tRNAs undergo post-transcriptional editing to correct mismatches occurring at the acceptor stem. Modifications are limited to the first three nucleotides of the 5’ half, with the 3’ half serving as a guide and template to restore standard base pairs [26,27]. Each type of transition/transversion is possible, with pyrimidine-to-purine transversion being the most common. Ledee and Byers [22] also reported purine-to-pyrimidine editing, not observed in *A. terricola*, in three genes (*trnP*, *trnD*, *trnM*) in various strains (*Acanthamoeba royreba*, Diamond group, T1, T3, T2, OX1). In the present analysis, purine-to-pyrimidine editing was observed in the same genes in various other strains, as well as in *trnI2* (*A. lugdunensis*) and *trnA* (Linc-Ap1, *Acanthamoeba* T13, *A. astronyxis*).

## 4. Discussion

Currently, *Acanthamoeba* strains are identified by sequencing 18S rDNA. For routine analyses, the complete sequence is too long, and the short 450-bp fragment ASA.S1 is often chosen instead. This fragment is amplified using genus-specific primers [28] and contains the 29-1 stem, which allows almost all genotypes to be distinguished. The use of mtSSU sequences is much less common, although a few previous studies have demonstrated their potential [6,8,9,10,11,29]. In various other amoebae, a fragment of the mitochondrial gene for cytochrome oxidase c subunit 1 (*cox1* or COI) is increasingly being used, as it increases the ability to distinguish between closely related lineages and may be useful in unravelling cryptic species [30,31,32]. The Cox1 barcoding strategy, initially developed for animals [33,34,35], has also been applied to *Acanthamoeba* [20,29], appearing to be more suitable than 18S rDNA for species delimitation. Indeed, both nuclear SSU and LSU rDNA sequences seem better suited to resolving deep relationships within *Acanthamoeba*, i.e., to recognising genotypes, while mtSSU allows, at least in the case of the T4 genotype, less deep nodes to be disentangled as subtypes. However, groups of strains that could represent natural assemblages, i.e., species, seem to be better identified by rapidly evolving sequences such as cox1. Additional markers with similar taxonomic depths would therefore be useful, given the persistent confusion, despite previous efforts, in strain/species assignment within *Acanthamoeba*.

The analysis of mitochondrial rDNA spacers in *Acanthamoeba* performed in this study yielded lineage-specific profiles consistent with those obtained from nuclear and mitochondrial SSU rDNA sequences [6,9]. This contradicts the previously observed lack of sequence alignment and the limited utility of this region [22], likely because a larger number of strains and genotypes were studied using more powerful software. A major difference was observed between MG1 and MG2/MG3 species in the arrangement of tRNA genes (Figure 1). This should be confirmed by additional data from other MG1 strains and species. If so, such a rearrangement could provide further evidence that MG1 forms a genus-level lineage distinct from other *Acanthamoeba* [5,6]. No data are available regarding the mitochondrial spacer of other Acanthamoebidae, while that of the more distantly related *Balamuthia mandrillaris* (Centramoebida, Balamuthiidae) differs in the presence of the cytochrome oxidase subunit 2 (*cox2*) gene, which separates the LSU from the SSU. A cluster of five other tRNA genes is located near the SSU [36]. In other amoebozoans for which mtDNA is available, the LSU and SSU rRNA genes are either close to each other or separated by a variable number of protein-coding genes and/or tRNAs, depending on the genus/species [37]. Consequently, at present, only *Acanthamoeba* is characterised by a mitochondrial rDNA spacer.

**Figure 3:**
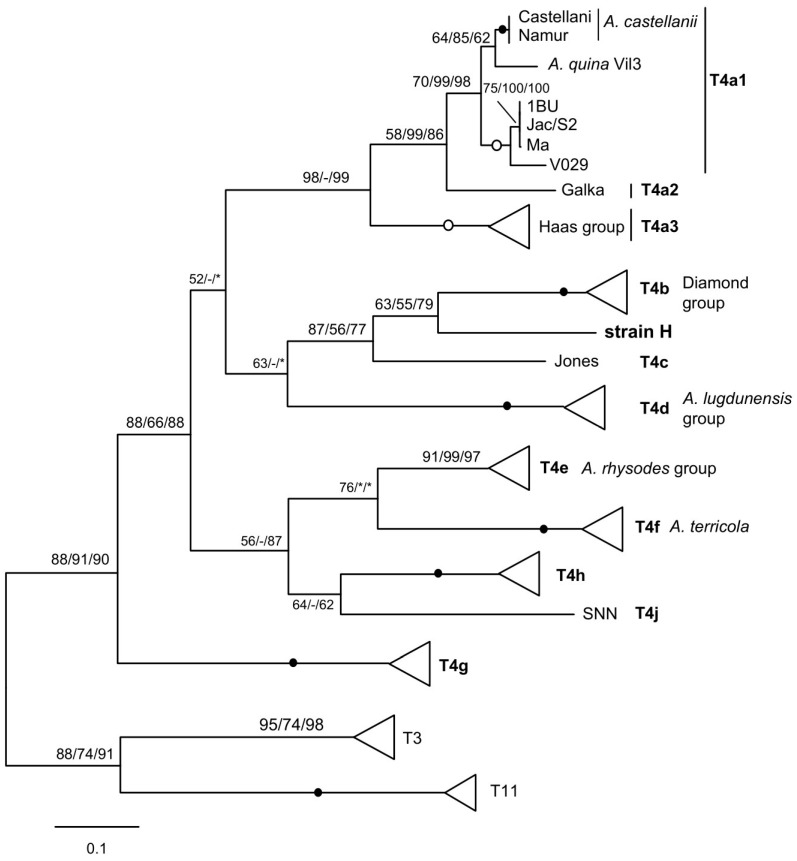
Molecular phylogeny of the mitochondrial rDNA spacer of T4 strains. The strain H representing a putative novel subtype is in bold. Spacers from T3 and T11 strains were used as an outgroup. The collapsed groups and symbols are as in Figure 2.

The MG2 and MG3 species analysed all have the same type of mitochondrial rDNA spacer as described previously [21,22]. Its seemingly insoluble extreme heterogeneity in length and sequence can in fact be coherently correlated with different mtSSU genotypes or subtypes, both by region size profiles (Table 1) and by molecular phylogenetic analysis (Figure 2). This is particularly convincing in the case of the numerous T4 sequences, where the distribution according to the mtSSU subtype was perfectly found. The mitochondrial rDNA spacer could therefore serve as an additional marker for molecular typing and phylogenetic analysis. As an example, another T4A strain currently under study, labelled H, indistinguishable from the others by its nuclear SSU rDNA, has an mtSSU sufficiently divergent to constitute a possible new subtype. Interestingly, the strain emerges as a distinct lineage when the mitochondrial rDNA spacer is analysed (Figure 3).

The region size profile of strain H is also unique, although very similar to those of the closely related Jones and the Diamond group, varying mainly in the *trnD*–*trnM* region. For this strain, the mtSSU and spacer consistently identify a new mitochondrial subtype, although further evaluation using other markers is required to confirm this with certainty. In any case, phylogenetic analysis of spacers seems promising for identifying the different groups within T4, which is the predominant genotype in the environment and the most common in clinical samples. The T4 genotype appears to be so rich and diverse that other SSU subtypes will likely be identified in the future. Solid natural groupings can be delineated by perfect nuclear and mitochondrial matches [6,8,20], using more appropriate markers such as *cox1* to unravel internal nodes, i.e., identify species. This is the case for almost all nuclear subtypes, with the exception of subtypes T4A and T4B, for which mitochondrial analysis reveals several subtypes and often mixed matches [6,29]. The mitochondrial rDNA spacer could be an additional marker, complementary to mtSSU and *cox1*, for analysing these relationships.

Targeting mitochondrial DNA to detect *Acanthamoeba* may have multiple advantages. It is estimated that growing trophozoites contain approximately 3300 mtDNA per cell [38], which is more than five times the amount of nuclear rDNA (~600 copies per cell) [39], the most commonly used target for identification. Even within cysts, the amount of mtDNA, tenfold reduced, remains important. Mitochondrial rRNA genes are easier to amplify and sequence than their nuclear counterparts, with similar results, and analysis of other genes, such as *cox1*, also shows good correlation with the current genotyping system [6,8,9,20,29]. The combined use of different nuclear and mitochondrial markers will certainly be useful for more reliable delineation of lineages and more accurate identification of strains.

## Figures and Tables

**Table 1:** Length differences (bp) between the tRNA genes in the mitochondrial LSU-SSU rDNA spacer region.

SSU GT^1^		Species/strain	Culture collection	Spacer length	LSU-I2	I2-A	A-P	P-D	D-M	M-SSU
mt	nucl									
T4a1	T4A	*A. castellanii* Castellani^2^	ATCC 50374	812	15	6	11	3	349	68
T4a1	T4A	*A. castellanii* Namur	na	815	15	6	11	3	352	68
T4a1	T4A	*A. quina* Vil3	ATCC 50241	799	19	6	9	2	343	60
T4a1	T4B	Asp Ma^2^	ATCC 50370	765	15	6	11	2	305	66
T4a1	T4B	Asp Jac/S2^2^	ATCC 50372	766	15	6	11	2	306	66
T4a1	T4B	Asp 1BU	ATCC PRA-105	766	15	6	11	2	307	66
T4a1	T4B	Asp CDC V029^2^	ATCC 50495	736	14	6	11	2	281	62
T4a2	T4A	Asp Galka^2^	ATCC 50496	847	15	7	11	2	378	74
T4a3	T4A	Asp Haas^2^	ATCC 50368	823	19	6	12	2	361	64
T4a3	T4A	Asp BCP	na	841	19	7	12	2	372	69
T4b	T4B	Asp Diamond^2^	ATCC 50724	635	14	6	17	2	161	75
T4b	T4B	Asp CDC V125^2^	ATCC 50498	636	14	6	19	2	160	75
T4b	T4B	Asp CDC 0180:1^2^	ATCC 50491	636	14	6	19	2	160	75
T4c	T4A	Asp Jones	ATCC 30461	597	14	9	14	2	125	73
T4d	T4A	*A. lugdunensis* L3a	ATCC 50240	885	15	8	11	3	427	61
T4d	T4A	Asp Linc-AP1	CCAP 1501/18	888	15	8	14	3	427	61
T4e	T4D	*A. royreba* Oak Ridge^2^	ATCC 30884	772	11	48	11	2	293	47
T4e	T4D	*A. rhysodes* Singh	ATCC 30973	773	11	48	11	2	295	47
T4e	T4D	*A. mauritaniensis* 1652	ATCC 50253	676	14	57	14	3	179	49
T4g	T4F	*A. triangularis* SH621	ATCC 50254	659	17	20	11	2	210	39
T4g	T4C	Asp SK_2022c	na	659	17	20	11	2	211	38
T4g	T4C	Asp Fernandez^2^	ATCC 50369	663	17	20	11	2	214	39
T4f	T4G	*A. terricola* Neff^3^	ATCC 30010	635	33	30	7	3	136	65
T4f	T4G	*A. terricola* TN	na	635	34	30	7	3	136	65
T4j	T4H	Asp SNN	na	547	36	42	9	3	42	55
T4h	T4E	Asp WBN	na	588	11	34	6	5	117	55
T4h	T4E	Asp DYH	na	587	11	34	6	5	116	55
T3	T3	*A. griffini* S7^2^	ATCC 30731	559	16	15	0	19	109	41
T3	T3	*A. griffini* H37	ATCC 50702	562	15	15	0	19	110	44
T3	T3	Asp YM	na	549	13	14	0	10	109	44
T3	T3	Asp Panola Mtn.^2^	ATCC 30487	611	14	14	0	10	143	71
T11	T11	Asp SK_2022a	na	1098	31	30	12	26	598	41
T11	T11	Asp SK_2022b	na	1099	31	30	12	26	599	41
T2	T2	*A. palestinensis* Reich^2^	ATCC 30870	1360	11	29	7	17	877 ^4^	59
OX1	OX1	Asp Sawyer OX1^2^	CCAP 1501/3c	1190	16	44	9	19	684	58
T1	T1	Asp CDC V006^2^	ATCC 50494	1288	47	39	–1	21	807	52
T13	T13	Asp UWET39	ATCC PRA-9	560	11	13	–3	8	116	56
T22	T22	Asp	na	645	35	11	3	13	165	59
T5	T5	*A. lenticulata* 72/2	ATCC 50704	484	15	5	0	20	8	77
T5	T5	*A. lenticulata* PT14	na	478	14	5	0	21	9	70

^1^ Genotypes (GT) based on mitochondrial (mt) and nuclear (nucl) SSU rDNA sequences; ^2^ strains previously analysed by Ledee and Byers [22] (sequences FJ411148–FJ411163); ^3^ reference mitochondrial genome [21]; ^4^ presence of duplicate *trnM* gene (see Ledee and Byers [22]). na: not available.

## Data Availability

The original contributions presented in this study are included in the article/supplementary material. Further inquiries can be directed to the corresponding author.

## Supplementary Materials

The following supporting information can be downloaded at https://www.mdpi.com/article/10.3390/microorganisms13102285/s1, Table S1: List of mitochondrial rDNA spacer of *Acanthamoeba* accession numbers.
