# Supplementary material for: Molecular Typing of Acanthamoeba Using Mitochondrial rDNA Spacers
Source: Microorganisms. 2025 Sep 30;13(10):2285. doi: 10.3390/microorganisms13102285 (PMC12566451; doi:10.3390/microorganisms13102285)
Supplement: Supplementary file 1 [file microorganisms-13-02285-s001.zip › microorganisms-3853160-supplementary.pdf]

Table S1. List of mitochondrial rDNA spacer of *Acanthamoeba* accession numbers

Genotype (nucl/mt)	species/strain	WSG project SRA run	acc. nos.
T4A/T4a1	*A. castellanii* Castellani		FJ411151
T4A/T4a1	*A. castellanii* Namur	CAIJLO01	CAIJLO010000313.1:c23536-22476
T4A/T4a1	*A. quina* Vil3	CDFN01	CDFN01059117.1:2951-5486 ; CDFN01027700.1
T4B/T4a1	Asp Ma		FJ411152
T4B/T4a1	Asp Jac/S2		FJ411153
T4B/T4a1	Asp CDC V029		FJ411154
T4AB/T4a1	Asp 1BU	JBJUIN01	JBJUIN010000474.1:c31719-30700
T4A/T4a2	Asp Galka		FJ411155
T4A/T4a3	Asp Haas		FJ411156
T4A/T4a3	Asp BCP		KT185628
T4B/T4b	Asp Diamond		FJ411148
T4B/T4b	Asp CDC V125		FJ411149
T4B/T4b	Asp CDC 0180:1		FJ411150
T4A/T4c	Asp Jones	SRX18334598	
T4A/T4d	*A. lugdunensis* L3a	CDFB01	CDFB01009997.1; CDFB01000550.1; CDFB01028947.1; CDFB01030069.1; CDFB01030880.1; CDFB01014059.1; CDFB01001725.1; CDFB01028540.1; CDFB01031739.1; CDFB01025392.1; CDFB01002601.1; CDFB01005223.1
T4A/T4d	Asp Linc-AP1		KP054475
T4D/T4e	*A. royreba* Oak Ridge		FJ411157
T4D/T4e	*A. rhysodes* Singh	CDFC01	CDFC01026872.1; CDFC01030119.1; CDFC01000876.1; CDFC01036600.1; CDFC01011793.1;CDFC01013172.1;CDFC01037396.1;CDFC01005444.1;CDFC01022386.1;CDFC01035927.1 ; CDFC01031097.1
T4D/T4e	*A. mauritaniensis* 1652	CDFE01	CDFE01062806.1; CDFE01020180.1 ; CDFE01022826.1 ; CDFE01019725.1
T4F/T4g	*A. triangularis* SH621	CDFD01	CDFD01022159.1;CDFD01009831.1;CDFD01053056.1
T4C/T4g	Asp SK_2022c	JANDKB01	JANDKB010006468.1:1312-2389
T4C/T4g	Asp Fernandez		FJ411158
T4G/T4f	*A. terricola* Neff		U12386
T4G/T4f	*A. terricola* TN		KX580904
T4E/T4h	Asp WBN	SRR18367196	
T4E/T4h	Asp DYH	SRR29455173	
T4H/T4j	Asp SNN	SRR18367192	
T3	*A. griffini* S7		FJ411159
T3	*A. griffini* H37	JBJUIM01	JBJUIM010000862.1:11895-12716
T3	Asp Panola Mtn.		FJ411160
T3	Asp YM	SRR18367191	
T11	Asp SK_2022a	JANEZP01	JANEZP010002006.1:9231-10790
T11	Asp SK_2022b	JANDJZ01	JANDJZ010001723.1:5799-7359
T2	*A. palestinensis* Reich		FJ411161
OX1	Asp Sawyer OX1		FJ411162
T1	Asp CDC V006		FJ411163
T13	Asp UWET39	JQAK01	JQAK01000007.1:c24000-23200
T22	Asp	CDEZ01	CDEZ01003962.1:c248-1 ; CDEZ01016229.1; CDEZ01003412.1; CDEZ01009627.1:c127-1; CDEZ01008734.1 ;CDEZ01022867.1; CDEZ01022824.1:c1486-1106
T5	*A. lenticulata* 72/2	MSTW01	MSTW01021796.1:c1016-1 ; MSTW01020807.1
T5	*A. lenticulata* PT14	NAVB01	NAVB01010535.1:c1398-844 ; NAVB01010519.1 :790-1022
T18	*A. byersi* Pb30/40	MRZZ01	MRZZ01044211.1:c1315-1
